# A Dual Luciferase Reporter System for *B. burgdorferi* Measures Transcriptional Activity during Tick-Pathogen Interactions

**DOI:** 10.3389/fcimb.2017.00225

**Published:** 2017-05-31

**Authors:** Philip P. Adams, Carlos Flores Avile, Mollie W. Jewett

**Affiliations:** Division of Immunity and Pathogenesis, Burnett School of Biomedical Sciences, University of Central Florida College of MedicineOrlando, FL, United States

**Keywords:** *Borrelia* (*Borreliella*) *burgdorferi*, Lyme disease, tick-pathogen interactions, bioluminescence reporter, *Photinus reniformis* luciferase, *Photinus pyralis* luciferase

## Abstract

Knowledge of the transcriptional responses of vector-borne pathogens at the vector-pathogen interface is critical for understanding disease transmission. *Borrelia* (*Borreliella*) *burgdorferi*, the causative agent of Lyme disease in the United States, is transmitted by the bite of infected *Ixodes sp*. ticks. It is known that *B. burgdorferi* has altered patterns of gene expression during tick acquisition, persistence and transmission. Recently, we and others have discovered *in vitro* expression of RNAs found internal, overlapping, and antisense to annotated open reading frames in the *B. burgdorferi* genome. However, there is a lack of molecular genetic tools for *B. burgdorferi* for quantitative, strand-specific, comparative analysis of these transcripts in distinct environments such as the arthropod vector. To address this need, we have developed a dual luciferase reporter system to quantify *B. burgdorferi* promoter activities in a strand-specific manner. We demonstrate that constitutive expression of a *B. burgdorferi* codon-optimized *Renilla reniformis* luciferase gene (*rluc*_*Bb*_) allows normalization of the activity of a promoter of interest when fused to the *B. burgdorferi* codon-optimized *Photinus pyralis* luciferase gene (*fluc*_*Bb*_*)* on the same plasmid. Using the well characterized, differentially regulated, promoters for flagellin (*flaBp*), outer surface protein A (*ospAp*) and outer surface protein C (*ospCp*), we document the efficacy of the dual luciferase system for quantitation of promoter activities during *in vitro* growth and in infected ticks. Cumulatively, the dual luciferase method outlined herein is the first dual reporter system for *B. burgdorferi*, providing a novel and highly versatile approach for strand-specific molecular genetic analyses.

## Introduction

Vector-borne illnesses account for 17% of worldwide infectious diseases, amounting to over one billion cases yearly (World Health Organization, 2016). Ticks are notorious for delivering a diversity of infectious agents to their hosts during the blood meal. Of these pathogens the *Borrelia burgdorferi* sensu lato complex or *Borreliella* genus (Adeolu and Gupta, 2014), the spirochete group that causes Lyme disease, contributes the highest incidence of arthropod-transmitted bacterial infection worldwide (Schotthoefer and Frost, 2015). Particular to the United States, *Borrelia* (*Borreliella*) *burgdorferi* interaction with and colonization of *Ixodes* species is highly specific (de Silva et al., 2009), with no other natural arthropod vector identified to date.

Newly hatched larval ticks are not colonized with *B. burgdorferi*, as there is currently no evidence to support transovarial transmission of the pathogen (Rollend et al., 2013). Rather, larvae can become infected by feeding on one of the numerous small vertebrates that serve as reservoirs for *B. burgdorferi* in nature, such as the white-footed mouse *Peromyscus leucopus*. Larval ticks then undergo an approximate month-long morphogenesis process and molt into nymphs. All the while, *B. burgdorferi* reside in the tick midgut. Like the larvae, the infected nymphs take a single blood meal from a vertebrate followed by morphogenesis to adults. During nymph feeding, *B. burgdorferi* migrate from the midgut to the tick salivary glands and are transmitted to the vertebrate host, maintaining the spirochete in its enzootic cycle (Radolf et al., 2012). Therefore, it has been proposed that *B. burgdorferi* undergoes three major tick-related events that require complex genetic regulation: acquisition, persistence, and transmission (Iyer et al., 2015; Caimano et al., 2016).

Survival of *B. burgdorferi* in the tick requires that the spirochete overcome a number of environmental stress conditions, such as starvation and assault from tick immune factors (Radolf et al., 2012; Caimano et al., 2016). Recently, open reading frame-based microarray analysis has provided insight into the gene expression changes that occur in the *B. burgdorferi* transcriptome in fed larvae, fed nymphs, and under mammalian host-like conditions in dialysis membrane chambers (Iyer et al., 2015). The unusual structure of *B. burgdorferi*'s genome and its scarcity of characterized transcription factors, further contribute to interest in understanding the mechanisms of stress adaptation and gene regulation that the spirochete employs during its interaction with the tick vector. The *B. burgdorferi* segmented genome, in characterized type strain B31, is composed of an approximate 900 kb linear chromosome and 21 plasmids of size ranges 5–56 kb that include many annotated open reading frames (ORFs) of unknown function (Fraser et al., 1997; Casjens et al., 2012). A recent global examination and 5′ end mapping of the *B. burgdorferi* transcriptome by our laboratory has revealed that the spirochete is rich with “overlapping transcripts” where 63% of total RNA species are transcribed internal and 13% antisense to annotated open reading frames (Adams et al., 2017). Other recent RNA-seq based applications have also described the presence of these transcripts in *B. burgdorferi* (Arnold et al., 2016; Popitsch et al., 2017). These findings are supported by similar analyses in other bacteria, which have revealed complex transcriptomes that include a variety of antisense, intragenic, intergenic, and orphan transcripts, which in some cases represent the majority of transcript types as opposed to mRNAs for annotated open reading frames (Sharma et al., 2010; Kroger et al., 2012; Thomason et al., 2015). These discoveries drive the need for the development of new molecular genetic tools for investigating the expression patterns and functional roles of novel RNA transcripts in a strand-specific manner.

For over three decades, researchers have been isolating, expressing, and adapting bioluminescence genes for biomedical applications (de Wet et al., 1987; Lorenz et al., 1991). These techniques are based on the enzymatic (i.e., luciferase) oxidation of a substrate (i.e., luciferin) to generate light. Transcriptional reporters using bioluminescence read-outs have proven to be robust and sensitive molecular tools for investigating transcript expression (Andreu et al., 2011). Infectious disease-based research has resulted in the development of multiple luciferase systems for a variety of pathogens, and demonstrated that relative luciferase units of constitutively expressed bioluminescence reporters correlate to bacterial numbers (Andreu et al., 2011). Advanced and high-throughput adaptations for transcriptional reporters utilize multiple luciferase enzymes with unique substrates, which are compatible within the same experimental setup. In this manner, one luciferase serves as an experimental readout of promoter activity and the other as the normalization control for cell number (McNabb et al., 2005; Wright et al., 2005). A previously engineered *B. burgdorferi* codon-optimized *Photinus pyralis* (firefly) luciferase gene (Blevins et al., 2007), when fused to a constitutive promoter, has been successful for *in vivo* live imaging of *B. burgdorferi* dissemination during mouse infection (Hyde et al., 2011; Wager et al., 2015). Furthermore, this luciferase reporter has been used to characterize the promoters for a variety of annotated ORFs and novel RNAs during *in vitro* cultivation, *in vivo* mouse infection, and in infected mouse tissues *ex vivo* (Skare et al., 2016; Adams et al., 2017). However, this reporter plasmid is limited in that it does not contain a constitutive control reporter to allow normalization and quantitation of the data. In order to expand the utility of this approach, we engineered a dual luciferase plasmid that carries both a constitutively expressed *B. burgdorferi* codon-optimized *Renilla reniformis* (sea pansy) luciferase gene and the *B. burgdorferi* codon-optimized *Photinus pyralis* (firefly) luciferase gene driven by a promoter of interest. Luciferin, the substrate of *Photinus pyralis* luciferase, emits yellow-green photons (550–570 nm) of light (Marques and Esteves da Silva, 2009), whereas coelenterazine, the substrate of *Renilla reniformis* luciferase, produces light in the blue spectrum (470 nm) (Woo et al., 2008). Functioning on the premise that each luciferase enzyme requires unique substrates for bioluminescence readout, this approach provides a method for quantitative measurement of strand-specific transcription, in an environment of interest. It has been previously demonstrated that coelenterazine-based luciferase reporters are ineffective for *in vivo* live imaging detection of bacterial pathogens during murine infection (Andreu et al., 2010), despite successful *in vivo* applications for mammalian tumor systems (Bhaumik and Gambhir, 2002). Herein, our studies demonstrate the efficacy of the *B. burgdorferi* dual luciferase system for genetic studies during *in vitro* cultivation of spirochetes and analysis of transcriptional activity that occurs at the tick-pathogen interface, which is critical for understanding the interactions of *B. burgdorferi* with the tick vector for the development of novel therapeutic strategies for Lyme disease.

## Materials and methods

### Bacterial strains and growth conditions

*B. burgdorferi* clones used in this study were derived from strain B31. For genetic manipulations infectious low-passage clone A3-68Δ*bbe02* was utilized, which lacks cp9, lp56, and gene *bbe02* on lp25 (Rego et al., 2011), and herein referred to as wild type. Spirochetes were cultivated in liquid Barbour-Stoenner-Kelly (BSK) II medium supplemented with gelatin and 6% rabbit serum (Barbour, 1984) and grown at 35°C with 2.5% CO_2_. Luciferase plasmids were engineered in DH5α *E. coli*, grown in LB broth or on LB agar plates containing 300 μg/ml spectinomycin when appropriate, and transformed into *B. burgdorferi* as previously described (Samuels, 1995). Transformants were selected by plating in solid BSKII medium as previously described (Rosa and Hogan, 1992), in the presence of 50 μg/ml streptomycin and/or 200 μg/ml kanamycin, when applicable. All transformants were verified by PCR to contain the plasmid content of the parent clone (Elias et al., 2002; Jewett et al., 2007).

### Construction of the dual luciferase plasmids

The *Renilla reniformis* luciferase gene (Lorenz et al., 1991) was codon-optimized for *B. burgdorferi* (*rluc*_*Bb*_) with the OptimumGene™ algorithm, synthesized, and cloned into the *E. coli* vector pUC18 (Genscript) (Genebank accession number MF043582). All primer sequences are listed in Table 1. The *rluc*_*Bb*_ gene was PCR amplified from pUC18-*rluc*_*Bb*_ plasmid DNA using Phusion High-fidelity DNA polymerase (NEB) and primer pair 1732 and 1733. This also resulted in the addition of 27 bp of DNA from the 3′ of the *flaB* promoter to the 5′ of *rluc*_*Bb*_. Concurrently, a DNA fragment containing the *flaB* promoter sequence with a 24 bp overhang from the 5′ of the *rluc*_*Bb*_ gene was Phusion-PCR amplified using B31 A3 genomic DNA and primer pair 1730 and 1731. The PCR fragments were ligated together by combining Gibson Assembly® Master Mix (NEB) and 0.16 pmol of each PCR fragment and incubating the reaction at 50°C for 1 h. One microliter of assembled product (*flaBp-rluc*_*Bb*_) was Phusion-PCR amplified using primers 1730 and 1733 and the DNA fragment gel extracted using the QIAquick Gel Extraction kit (Qiagen), and cloned into pCR-Blunt using the Zero Blunt PCR cloning kit (Invitrogen) according to the manufacturer's instructions. The sequence of the *flaBp-rluc*_*Bb*_ cassette was verified by Sanger sequencing.

**Table 1:** Oligonucleotide primers used in this study.

**Number**	**Name**	**Sequence (5′-3′)**
1730	*flaBp* 5′	TGTCTGTCGCCTCTTGTG
1731	*flaBp* 3′, 24 bp overlay *rlucBb* 5′	AGGATCATAAACTTTACTTGTCATGATTGATAATCATATATCATTCCTCCA
1732	*rlucBb* 5′, 27 bp overlay *flaBp* 3′	TGGAGGAATGATATATGATTATCAATCATGACAAGTAAAGTTTATGATCCT
1733	*rlucBb* 3′	TTATTGTTCATTTTTCAATACTCGT
1850	*rlucBb* 3′ KpnI	CTAAGGTACCTTATTGTTCATTTTTCAATACTCGTTC
1910	*flaBp* 5′ BamHI	TGGCCGGATCCTGTCTGTCGCCTCTTGTGGC

The *flaBp-rluc*_*Bb*_ cassette was Phusion-PCR amplified from the pCR-Blunt *flaBp-rluc*_*Bb*_ plasmid using primer pair: 1850 and 1910, introducing BamHI and KpnI restriction sites. *B. burgdorferi* shuttle vectors containing the promoterless *B. burgdorferi* optimized *Photinus pyralis* luciferase gene (*fluc*_*Bb*_), *flaBp-fluc*_*Bb*_, *ospAp-fluc*_*Bb*_, or *ospCp-fluc*_*Bb*_ (Blevins et al., 2007; Adams et al., 2017) were digested with BamHI and KpnI high fidelity enzymes (NEB), gel extracted using the QIAquick Gel Extraction kit (Qiagen), and ligated to the BamHI/KpnI-digested *flaBp-rluc*_*Bb*_ cassette using T4 DNA ligase (NEB), generating plasmids pCFA701, pCFA801, pCFA802, and pCFA803. All plasmid constructs were confirmed by PCR, restriction digest, and Sanger sequencing.

### *In vitro* dual luciferase assay

*B. burgdorferi* clones were grown to logarithmic phase (3–7 × 10^7^ spirochetes/ml) or stationary phase (1–1.2 × 10^8^ spirochetes/ml) in 15 ml of BSKII medium and pelleted at 3,210 × g for 10 min. Cells were washed with phosphate buffered saline (PBS) (137 mM NaCl, 2.7 mM KCl, 10 mM Na_2_HPO_4_, 1.8 mM KH_2_PO_4_, pH 7.4) and resuspended in 300 μl of PBS. Eighty microliters of each sample was used to measure the optical density at 600 nm (OD_600_) using a BioTek Synergy 4. This resulted in an average OD_600_ value of ~0.25 for logarithmic phase spirochetes and ~0.36 for stationary phase spirochetes. One hundred microliters of each sample was loaded into a black, solid bottom 96-well plate (Corning) and combined with 700 μM D-luciferin (Regis) in PBS or 3.5 mM water soluble native coelenterazine (NanoLight Technology) in PBS. For samples containing coelenterazine, one well was left empty, in all directions around each sample, to decrease signal overlap between samples. For determining *B burgdorferi Photinus* luciferase (Fluc_Bb_) and *Renilla* luciferase (Rluc_Bb_) sensitivity, spirochetes containing pCFA801 were grown to logarithmic phase in 15 ml of BSKII medium, cell density determined using a Petroff Hauser counting chamber, washed with PBS, and resuspended in PBS to a density of 2 × 10^6^ cells/μl. Samples were serial diluted 10-fold and 100 μl of each dilution was loaded into a black, solid bottom 96-well plate (Corning) and combined with 700 μM D-luciferin or 3.5 mM coelenterazine. The relative luciferase units (RLUs) for Fluc_Bb_ and Rluc_Bb_ were determined by measuring photon emission in each well for 1 s, 10 times using the EnVision 2104 Multilabel Reader (PerkinElmer), following the addition of luciferin or coelenterazine substrate, respectively. Background relative Fluc_Bb_ or Rluc_Bb_ units, the average RLUs of the PBS control for either substrate, was subtracted from all experimental measurements, as appropriate. Background-subtracted relative Fluc_Bb_ units were then normalized to the OD_600_ value or 10^8^ background-subtracted relative Rluc_Bb_ units of the same sample, when applicable (e.g., 4 × 10^4^ Fluc_Bb_ units/0.1 OD_600_ value = 4 × 10^5^ relative Fluc_Bb_ units/OD_600_; 4 × 10^4^ Fluc_Bb_ units/0.06 10^8^ Rluc_Bb_ units = 6.4 × 10^5^ relative Fluc_Bb_ units/10^8^ Rluc_Bb_ units). The limit of detection (LoD) and quantification (LoQ) for Fluc_Bb_ and Rluc_Bb_ were established as the average RLUs for PBS alone plus 3 or 10 standard deviations, respectively. All experiments were conducted in biological triplicate.

### Ethics statement

The University of Central Florida is accredited by the International Association for Assessment and Accreditation of Laboratory Animal Care. Protocols for all animal experiments were prepared according to the guidelines of the National Institutes of Health and were reviewed and approved by the University of Central Florida Institutional Animal Care and Use Committee.

### *B. burgdorferi* infection of ticks

One week prior to inoculation, and throughout the duration of the study, mice were treated with 5 mg/ml streptomycin and 1 mg/ml Equal® sweetener in their water to maintain selection for the luciferase plasmid in the *B. burgdorferi* clones. Using *B. burgdorferi* carrying pJSB175, pCFA701, pCFA801, pCFA802, or pCFA803, groups of two 6–8 week old female C3H/HeN mice (Envigo) per clone were inoculated with 1 × 10^5^ spirochetes per mouse 80% intraperitoneally and 20% subcutaneously. The inoculum doses were verified by colony forming unit (CFU) counts in solid BSKII medium. All inoculum were PCR verified to contain the endogenous *B. burgdorferi* plasmids of the parent clone as previously described (Elias et al., 2002; Jewett et al., 2007). Three weeks post inoculation mouse infection was confirmed by positive seroreactivity against *B. burgdorferi* protein lysate as previously described (Schwan et al., 1989; Jewett et al., 2007). Groups of approximately 200 naïve *Ixodes scapularis* larvae each (Centers for Disease Control, BEI resources) were fed to repletion on the *B. burgdorferi* infected mice (Jewett et al., 2009). Mice were further confirmed for infection by reisolation of spirochetes from bladder and joint tissues, as described (Showman et al., 2016). Larvae were analyzed for infection (Grimm et al., 2005; Jewett et al., 2009). Briefly, ticks were individually surface sterilized by sequential washes in 100 μl of 3% H_2_O_2_, 70% ethanol, and sterile H_2_O. Subsets of larvae were analyzed for infection by reisolation of spirochetes in BSKII medium containing RPA cocktail (60 μM rifampicin, 110 μM phosphomycin, and 2.7 μM amphotericin B), immediately post feeding to repletion. Approximately 2 weeks following feeding, additional subsets of larvae were crushed and plated in solid BSKII containing RPA cocktail and 50 μg/ml streptomycin to determine CFU counts/larva. The remaining larvae were maintained and allowed to molt into nymphs. Two groups of 10–18 infected nymphs per *B. burgdorferi* clone were fed to repletion on naïve 6–8 week old female C3H/HeN mice (Envigo). These mice were treated with 5 mg/ml streptomycin and 1 mg/ml Equal® sweetener in their water 1 week prior to the feeding, to help sustain the luciferase plasmids in *B. burgdorferi* within the feeding nymphs. Throughout the duration of the study, ticks were stored in glass desiccation jars containing saturated potassium sulfate for to maintain appropriate humidity.

### *In vivo* tick dual luciferase assay

Approximately 2 weeks post feeding to repletion triplicate groups of 24 fed larvae or 8 fed nymphs per *B. burgdorferi* clone were crushed with a sterile pestle in 250 μl of PBS to generate tick extracts. For tick extracts, which were also plated for CFU counts, the ticks were first surface sterilized as described above, with a final wash in sterile PBS instead of H_2_O. Tick debris was allowed to settle and 100 μl of sample was removed and incubated with 700 μM D-luciferin (Regis) in PBS or 3.5 mM water soluble native coelenterazine (NanoLight Technology) in PBS. RLUs were measured as described for *in vitro* grown spirochetes. The limit of quantification (LoQ) for Fluc_Bb_ was established as the average relative Fluc_Bb_ units for PBS alone plus 10 standard deviations. The LoQ for Rluc_Bb_ was established as the average relative Rluc_Bb_ units for infected tick extracts with spirochetes containing pJSB175, which lacks the *rluc*_*Bb*_ gene, plus 10 standard deviations. Samples with relative Fluc_Bb_ units below the LoQ were given a value of zero; whereas, samples with relative Rluc_Bb_ units below the LoQ were removed from the analysis. Relative Fluc_Bb_ units were normalized to 10^8^ relative Rluc_Bb_ units for each sample. One microliter of each fed nymph extract was also plated for CFUs in solid BSKII containing RPA cocktail and 50 μg/ml streptomycin.

### Statistical analysis

GraphPad Prism version 7.02 was used for all statistical analyses. One-way ANOVA was used for analysis of all luciferase assays. For statistical comparison of the relative Fluc_Bb_ units normalized to OD_600_ or 10^8^ relative Rluc_Bb_ units, which had an extremely wide distribution (~10^1^–10^7^), all values were first square root transformed prior to statistical analysis. Following ANOVA, all samples were compared to the *B. burgdorferi* clones carrying the promoterless *fluc*_*Bb*_ control plasmid pJSB161 or pCFA701 using Dunnett's multiple comparison test. To compare two groups (i.e., the same clone in logarithmic versus stationary phase) following ANOVA, Bonferroni's multiple comparison test was applied to determine significance. For association analysis, Pearson correlation coefficient (r) was determined. *p* ≤ 0.05 was considered statistically significant for all statistical tests.

## Results

### Generation of the *B. burgdorferi* dual luciferase plasmid

**Figure 1:**
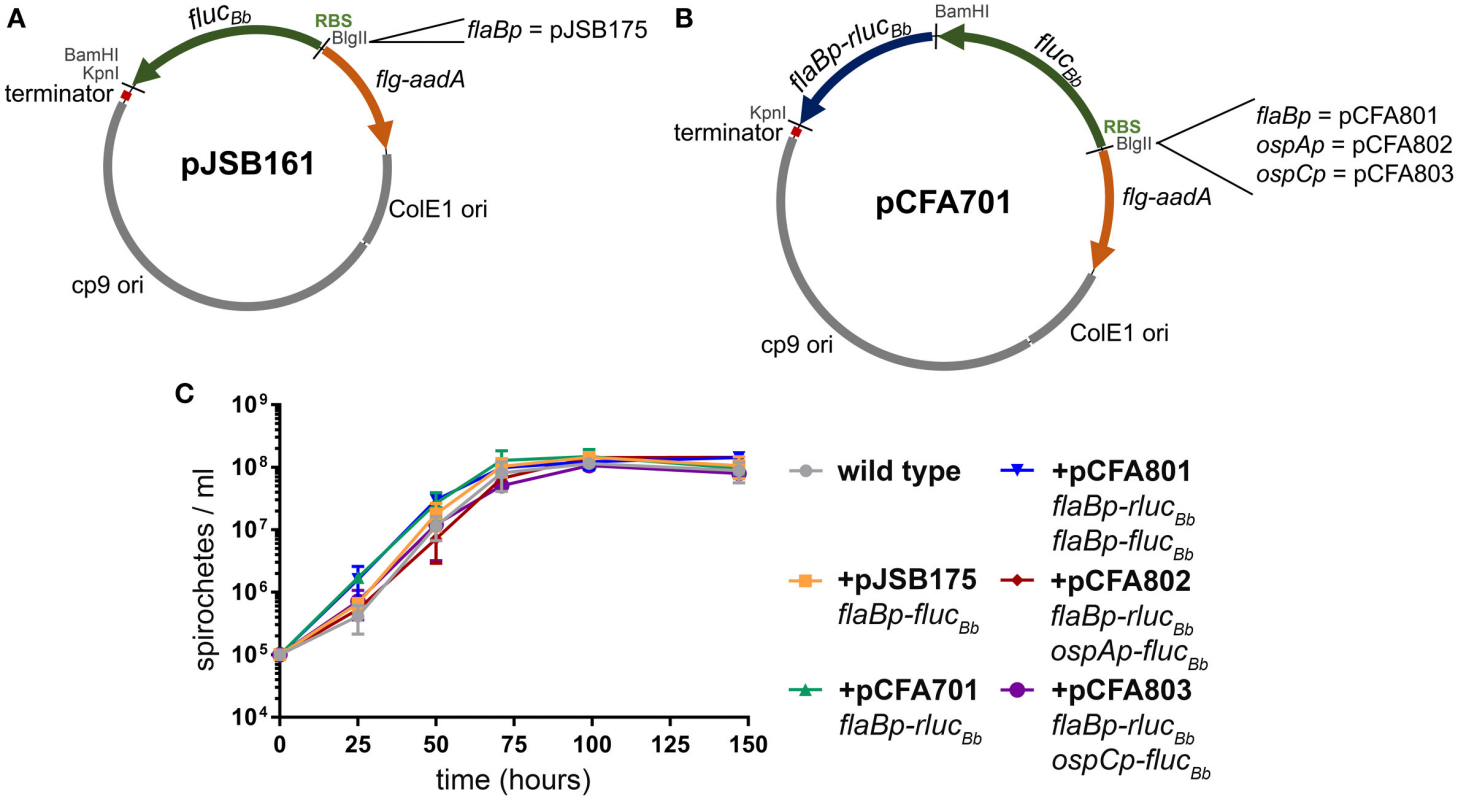
*B. burgdorferi* luciferase plasmids. All of the *B. burgdorferi* luciferase shuttle vectors were derived from pJSB161, which contains a Rho-independent transcription terminator sequence (terminator); ORFs 1, 2, and 3 of the *B. burgdorferi* cp9 replication machinery (cp9 ori); *E. coli* origin of replication (ColE1 ori); and the spectinomycin/streptomycin resistance cassette (*flg-aadA*) (Blevins et al., 2007). **(A)** The *B. burgdorferi* shuttle vector pJSB161 features a promoterless, *B. burgdorferi* codon optimized *Photinus pyralis* luciferase (*fluc*_*Bb*_), an upstream ribosome binding site (RBS) and a unique BlgII restriction site (Blevins et al., 2007). The plasmid pJSB175 was generated by addition of the *flaBp* promoter upstream of *fluc*_*Bb*_ in pJSB161 (Blevins et al., 2007). **(B)** The *B. burgdorferi* codon optimized *Renilla reniformis* luciferase (*rluc*_*Bb*_) gene under the control of the *flaB* promoter (*flaBp-rluc*_*Bb*_) was added to pJSB161, generating the *B. burgdorferi* dual luciferase shuttle vector, pCFA701. Plasmids, pCFA801, pCFA802, and pCFA803, harbor the *flaB, ospA*, and *ospC* promoters, respectively, upstream of *fluc*_*Bb*_. **(C)** The density of *B. burgdorferi* clone A3-68Δ*bbe02* (wild type) alone or harboring various *B. burgdorferi* luciferase plasmids was assessed over a period of 144 h using a Petroff Hauser counting chamber and dark-field microscopy. The data are presented as the mean spirochete density (spirochetes/ml) ± standard deviation over time (hours).

The *B. burgdorferi* shuttle vector pJSB161 (Blevins et al., 2007) contains a promoterless *B. burgdorferi* codon-optimized *Photinus pyralis* luciferase gene (*fluc*_*Bb*_*)* with a BlgII restriction site upstream of a ribosome binding site (RBS) for *fluc*_*Bb*_ (Figure 1A). This reporter plasmid allows a cloned promoter of interest to be analyzed for activity in a strand-specific manner via a bioluminescence detection method (Blevins et al., 2007; Skare et al., 2016; Adams et al., 2017). However, this approach does not allow for quantitative comparative analysis of promoter activity in different environments or between multiple promoters in the same environment due to the lack of an endogenous means to control for spirochete number across samples and conditions. Therefore to improve upon this technique for quantitative applications, we engineered a dual luciferase reporter system to constitutively express *Renilla reniformis* luciferase (*rluc*) (Lorenz et al., 1991), while maintaining *fluc*_*Bb*_ for quantifying the activity of a promoter of interest. Codon usage in *B. burgdorferi* is biased (Fraser et al., 1997; Nakamura et al., 2000), as the A/T nucleotide frequency is at 71.8% across the genome (Fraser et al., 1997; Adams et al., 2017). Codon optimization has been shown to improve production and activity of non-*B. burgdorferi* proteins expressed in *B. burgdorferi* (Blevins et al., 2007; Hayes et al., 2010). Therefore to prevent rare codons interfering with the *Renilla* luciferase reporter, the OptimumGene™ algorithm (GenScript) was used to refine the codon adaption index (CAI) of *rluc* (Lorenz et al., 1991) for *B. burgdorferi* from 0.64 to 0.85 (where a CAI value of 1.0 indicates the highest proportion of the most abundant codons) and synthesized (GenScript). This codon-optimized *rluc* gene (*rluc*_*Bb*_) (Genebank accession number MF043582) was cloned into pJSB161 (Blevins et al., 2007), for use in the dual luciferase reporter system under control of the constitutive promoter *flaBp* and corresponding ribosome binding site, generating pCFA701 (Figure 1B).

*B. burgdorferi* survival in the tick vector is essential for maintenance of the pathogen in its enzootic cycle. The spirochete is known to change its transcriptional profile at different stages of tick colonization including: acquisition, persistence during the molt, and transmission to the mammalian host (Iyer et al., 2015; Caimano et al., 2016). Because of our interest in applying the dual luciferase reporter system to quantitative analysis of *B. burgdorferi* promoter activities in the tick, we selected three well characterized promoters with distinct patterns of expression in the tick environment for proof of principle analysis. The flagellar protein promoter, *flaBp*, is constitutively active (Ge et al., 1997). The promoter for outer surface protein A (*ospAp*) is active during *in vitro* culture and in the tick during acquisition and persistence. In contrast, the promoter for outer surface protein C (*ospCp*) is active in the feeding tick during transmission and the mammalian host during the early stages of infection (Schwan et al., 1995; Schwan and Piesman, 2000; Schwan, 2003; Srivastava and de Silva, 2008). The *flaBp-rluc*_*Bb*_ cassette was cloned into three previously constructed plasmids, each containing one of these promoters driving the expression of *fluc*_*Bb*_ (Blevins et al., 2007; Adams et al., 2017), generating plasmids pCFA801, pCFA802, and pCFA803, respectively (Figure 1B). Spirochetes carrying pCFA701, pCFA801, pCFA802, or pCFA803 had no observed *in vitro* growth defect in BSKII medium compared to the wild type or *B. burgdorferi* carrying *flaBp-fluc*_*Bb*_ alone (pJSB175) (Blevins et al., 2007, Figure 1C).

### Rluc_Bb_ selectivity and limit of quantification

**Figure 2:**
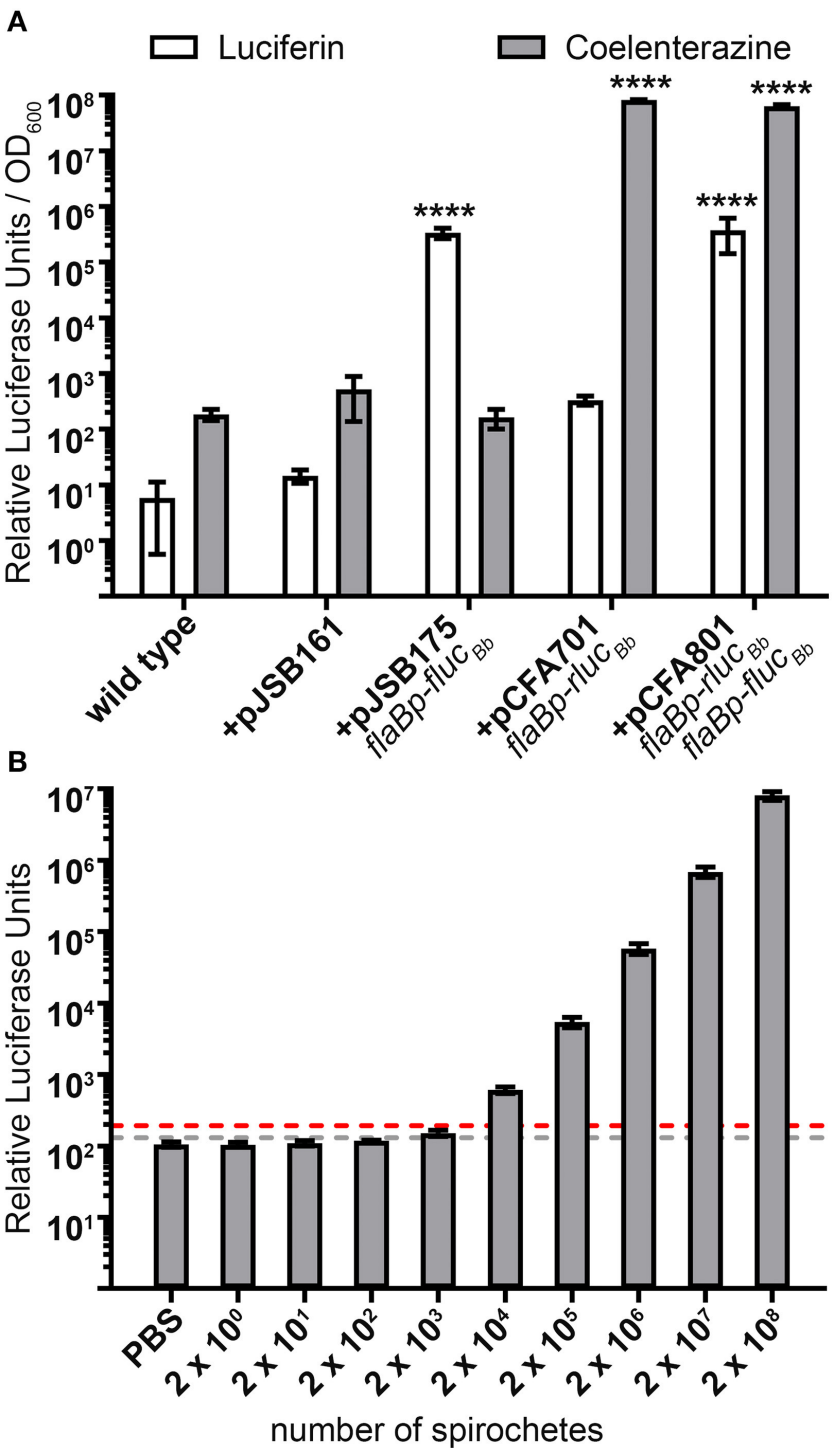
Selectivity and sensitivity of the dual luciferase assay in *B. burgdorferi*. **(A)** *B. burgdorferi* clones were grown to mid-logarithmic phase, and the *in vitro* luciferase assay performed with 700 μM D-luciferin or 3.5 mM coelenterazine. Relative luciferase units were normalized to optical density at 600 nm (OD_600_) and presented as the mean relative luciferase units/OD_600_ ± standard deviation for biological triplicate samples. The data were square root transformed and analyzed with a one-way ANOVA followed by Dunnett's multiple comparison test compared to *B. burgdorferi* containing the promoterless *fluc*_*Bb*_ (+pJSB161) for each substrate. Unless indicated, means were not significantly different from the control. Significant differences are indicated with asterisks (^****^p ≤ 0.0001). **(B)** Mid-logarithmic phase grown *B. burgdorferi* expressing both *flaBp-rluc*_*Bb*_ and *flaBp-fluc*_*Bb*_ (+pCFA801) were serial diluted from 2 × 10^8^ to 2 × 10^0^ spirochetes, and incubated with 3.5 mM coelenterazine. The limit of detection (LoD) was established as the mean relative luciferase units for PBS alone plus 3 standard deviations (gray dotted line). The limit of quantitation (LoQ) was established as the mean relative luciferase units for PBS alone plus 10 standard deviations (red dotted line). Data are presented as the mean relative luciferase units ± standard deviation for biological triplicate samples.

*Photinus pyralis* luciferase (Fluc) and *Renilla reniformis* luciferase (Rluc) are compatible for a dual reporter due to the specificity of each enzyme for distinct substrates (Bhaumik and Gambhir, 2002; McNabb et al., 2005). Therefore, we verified the selectivity of the Fluc_Bb_ and Rluc_Bb_ enzymes to recognize luciferin and coelenterazine, respectively. Based on our previous work using the *flaBp-fluc*_*Bb*_ reporter (Adams et al., 2017), we performed these analyses with approximately 3 × 10^8^ spirochetes harvested during log phase growth. As expected, the negative control, spirochetes not expressing *fluc*_*Bb*_ or *rluc*_*Bb*_ (+pJSB161), demonstrated no significant relative luciferase units for either substrate compared to wild type. Spirochetes expressing *fluc*_*Bb*_ alone (+pJSB175) demonstrated robust activity when incubated with luciferin, but no significant activity above the background of *B. burgdorferi* carrying pJSB161 when exposed to coelenterazine (Figure 2A). Conversely, spirochetes expressing *rluc*_*Bb*_ alone (+pCFA701) demonstrated strong activity when incubated with coelenterazine, but no significant activity above the negative control background when exposed to luciferin (Figure 2A). Spirochetes which express both *fluc*_*Bb*_ and *rluc*_*Bb*_ (+pCFA801) demonstrated significant relative luciferase units compared to spirochetes containing pJSB161 for both luciferin and coelenterazine. The background relative luciferase units for wild type and negative control spirochetes exposed to coelenterazine were found to be approximately 10-fold higher than those of the same spirochetes incubated with luciferin. Collectively, these data validated the ability of the codon-optimized Rluc_Bb_ enzyme to effectively oxidize coelenterazine and confirmed the specificity of the Fluc_*Bb*_ and Rluc_Bb_ enzymes for their respective substrates.

The utility of the dual luciferase reporter system not only depends on the substrate specificity of Fluc_Bb_ and Rluc_Bb_, but also the sensitivity of detecting and quantifying spirochetes expressing *rluc*_*Bb*_. The limit of detection (LoD) and limit of quantification (LoQ) were established as the number of spirochetes required to achieve relative Rluc_Bb_ units greater than that of phosphate-buffered saline (PBS) alone plus three standard deviations and 10 standard deviations, respectively. Analysis of triplicate samples of 10-fold serially diluted spirochetes, 2 × 10^8^ to 2 × 10^0^, harvested during log phase growth and incubated with coelenterazine, demonstrated 2 × 10^3^ spirochetes to be the lowest detectable number of *flaBp-rluc*_*Bb*_ expressing spirochetes in the assay (Figure 2B). However, the LoQ fell between 2 × 10^3^ and 2 × 10^4^ spirochetes. Saturation of the bioluminescence signal was never reached under the conditions examined, with a linear increase in relative Rluc_Bb_ units from 2 × 10^3^ to 2 × 10^8^ spirochetes (*y* = 0.0404x; *R*^2^ = 0.9997). Extrapolating from this linear equation, the LoQ was calculated to be 4.8 × 10^3^ spirochetes. These data indicate that a minimum of ~1 × 10^4^ *flaBp-rluc*_*Bb*_ expressing spirochetes are needed to achieve quantifiable relative Rluc_Bb_ units in the assay. Similar to what has been reported previously (Hyde et al., 2011), 2 × 10^3^ spirochetes was also found to be the lowest detectable number of *flaBp-fluc*_*Bb*_ expressing spirochetes (data not shown).

### The *flaBp-rluc_*Bb*_* reporter is a robust constitutive control for measuring *B. burgdorferi* promoter activities during *In vitro* growth

**Figure 3:**
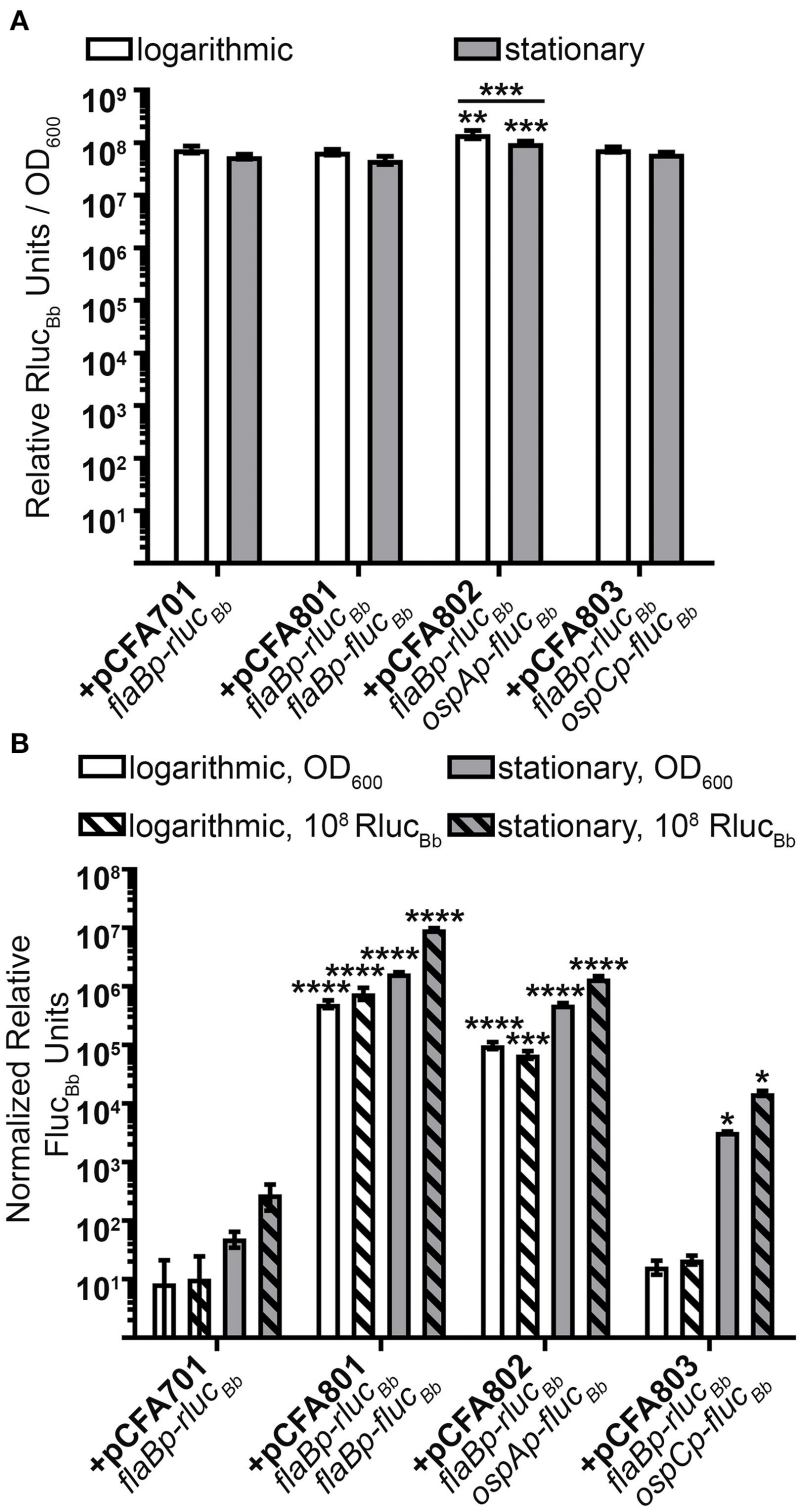
Dual luciferase assay with *in vitro* grown *B. burgdorferi*. Spirochetes were grown to either mid-logarithmic or stationary phase and the *in vitro* luciferase assay performed with 700 μM D-luciferin or 3.5 mM coelenterazine. All data are presented as mean normalized relative luciferase units ± standard deviation for biological triplicate samples. **(A)** Relative Rluc_Bb_ units normalized to OD_600_. The data set for each growth condition was analyzed with a one-way ANOVA followed by Dunnett's multiple comparison test compared to *B. burgdorferi* expressing *flaBp-rluc*_*Bb*_ (+pCFA701) and Bonferroni's multiple comparison test to compare the same clone in the two growth phases. **(B)** Relative Fluc_Bb_ units normalized to OD_600_ or 10^8^ relative Rluc_Bb_ units of the same sample. Each data set was square root transformed and analyzed with a one-way ANOVA followed by Dunnett's multiple comparison test compared to *B. burgdorferi* expressing *flaBp-rluc*_*Bb*_ (+pCFA701). Unless indicated, means were not significantly different from the control. Significant differences are indicated with asterisks (^*^p ≤ 0.05; ^**^p ≤ 0.01; ^***^p ≤ 0.001; ^****^p ≤ 0.0001).

Previously, we reported quantification of *in vitro* active *B. burgdorferi* promoters by normalizing relative luciferase units (RLUs) from *fluc*_*Bb*_ expressing cells to the optical density of the bacterial sample measured at 600 nm (OD_600_) (Adams et al., 2017). In this manner, the OD_600_ measurement reflects the number of spirochetes in the sample allowing normalization of RLUs across samples and assay conditions. To establish the *flaBp-rluc*_*Bb*_ reporter as an effective alternative for OD_600_ readings in our assay, first, relative Rluc_Bb_ units were measured for all *rluc*_*Bb*_-expressing *B. burgdorferi* clones and normalized to the number of spirochetes in the assay by OD_600_ (Figure 3A). All clones demonstrated consistent, robust relative Rluc_Bb_ units, ranging from 5 × 10^7^ to 1.68 × 10^8^. There was no significant difference among clones except for *B. burgdorferi* carrying pCFA802, which demonstrated higher relative Rluc_Bb_units/OD_600_ compared to all other clones as well as a difference between logarithmic and stationary phase growth. The same *rluc*_*Bb*_-expressing clones, were also incubated with luciferin and relative Fluc_Bb_ units were determined by normalizing to OD_600_ (Figure 3B). All *fluc*_*Bb*_ promoter fusions displayed the expected relative Fluc_Bb_ units/OD_600_, given the known expression patterns of their corresponding mRNA during logarithmic and stationary phase growth (Arnold et al., 2016). Both the *flaB* (+pCFA801) and *ospA* (+pCFA802) promoters demonstrated significant activity above the promoterless *fluc*_*Bb*_ control (+pCFA701) for both logarithmic and stationary phase growth. The activity of the *ospC* promoter (+pCFA803) during logarithmic phase growth was no different than the promoterless *fluc*_*Bb*_ control (+pCFA701). Whereas, the *ospC* promoter activity underwent significant induction from logarithmic to stationary phase growth (Figure 3B). Normalization of the relative Fluc_Bb_ units to 10^8^ relative Rluc_Bb_ units for each clone demonstrated no difference in the trend of the data resulting from this method of analysis compared to the data resulting from Fluc_Bb_ units normalized to OD_600_ (Figure 3B). Together these findings establish *flaBp*-*rluc*_*Bb*_ as an effective constitutive control reporter, whose quantitation is reflective of spirochete number and is a robust means to normalize data obtained from *fluc*_*Bb*_ promoter fusions using the dual luciferase reporter system.

### The dual luciferase reporter system quantifies promoter activities during tick-spirochete interactions

**Figure 4:**
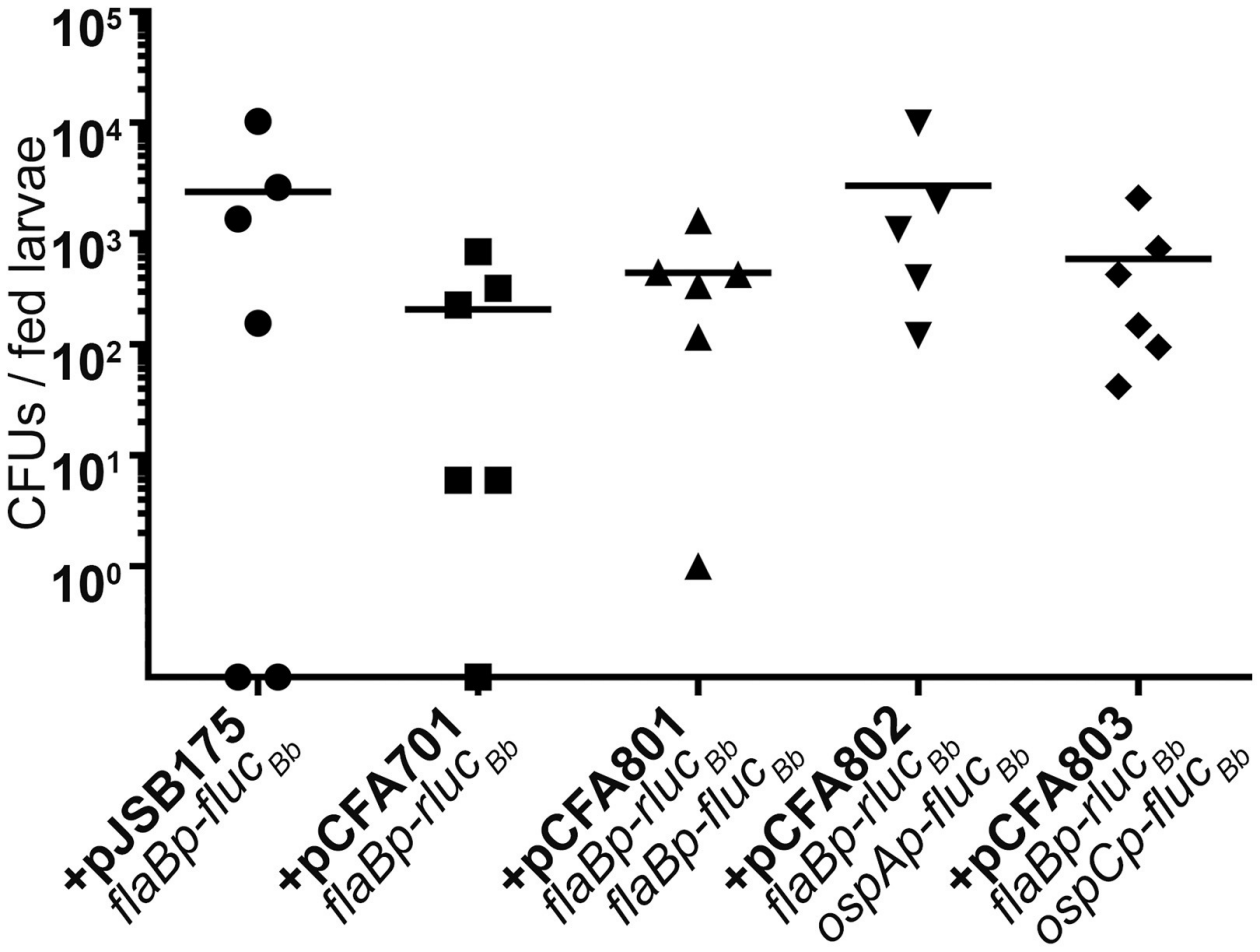
The dual luciferase plasmids do not affect *B. burgdorferi* acquisition efficiency. Groups of ~200 naïve *Ixodes scapularis* larvae were fed to repletion on mice infected with *B. burgdorferi* containing distinct luciferase plasmids. Subsets of individual fed larvae per *B. burgdorferi* clone were surface sterilized, crushed, and plated in solid BSKII containing RPA cocktail and 50 μg/ml streptomycin. Data points represent the number of colony forming units (CFUs) per individual fed larva. Uninfected larvae (CFU = 0) are represented as data points on the X-axis. No significant differences were detected across the data as determined with a one-way ANOVA.

Having established the dual luciferase reporter system for use with *in vitro* grown spirochetes, we examined the efficacy of the reporter system for measuring *B. burgdorferi* promoter activities in the tick vector. Naïve *Ixodes scapularis* larval ticks were infected with *B. burgdorferi* carrying the dual luciferase reporter plasmids or *flaBp*-*fluc*_*Bb*_, lacking *rluc*_*Bb*_ (+pJSB175) by feeding on groups of mice infected with the reporter clones via needle inoculation. Immediately following feeding to repletion, the percent of infected larvae per experimental group was determined by spirochete reisolation in BSKII medium. This analysis revealed that 60–90% of each experimental group of larvae successfully acquired *B. burgdorferi* upon feeding on infected mice. As an additional means to determine the percentage of infected larvae and to quantitate the number of spirochetes per tick, individual fed larvae were crushed and plated in solid medium for colony forming units (CFUs). Similar to the spirochete reisolation analysis, the groups of fed larvae were found by CFU analysis to be 66–100% infected. Moreover, although a broad range of spirochetes per tick was detected, there was no statistical difference between the average spirochete load per tick for each of the *B. burgdorferi* clones (Figure 4). These data suggest that all of the clones were able to colonize the ticks with the same efficiency.

**Figure 5:**
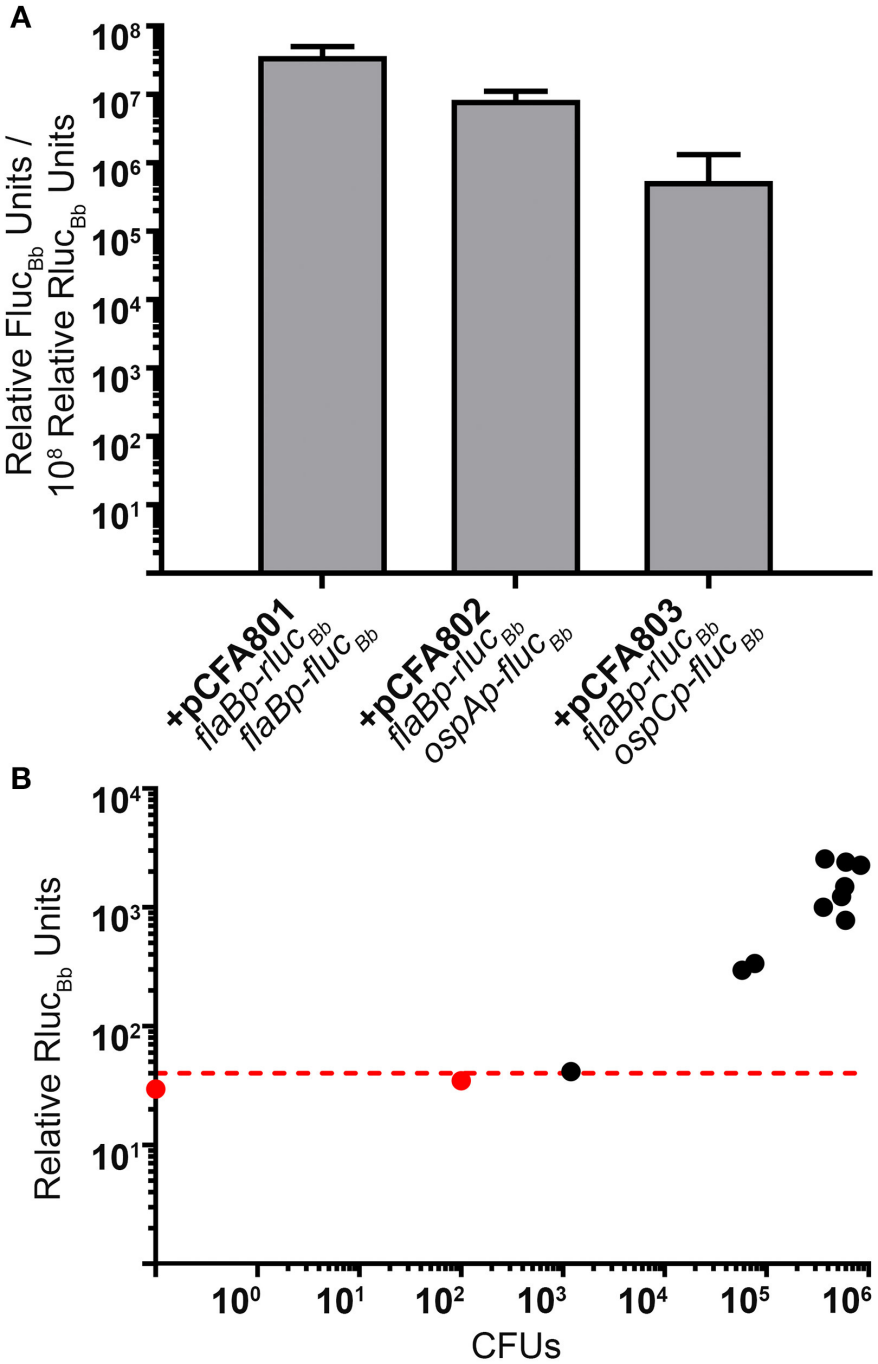
The dual luciferase assay quantitates known patterns of *B. burgdorferi* transcript expression in nymphs. **(A)** Groups of eight fed nymphs per *B. burgdorferi* clone were surface sterilized and crushed in 250 μl of PBS and the *in vivo* tick luciferase assay performed with 100 μl of tick extract and 700 μM D-luciferin or 3.5 mM coelenterazine. The data are presented as the mean relative Fluc_Bb_ units per 10^8^ relative Rluc_Bb_ units ± standard deviation (*n* = 3; *B. burgdorferi* carrying pCFA803 *n* = 2). **(B)** Relative Rluc_Bb_ luciferase activity is reflective of spirochete numbers in extracts from fed infected nymphs. 1 μl of fed nymph extract was plated for CFUs in solid BSKII containing RPA cocktail and 50 μg/ml streptomycin. The number of CFUs/100 μl of tick extract (CFUs) was plotted against the relative Rluc_Bb_ units for the same extract. The red dotted line indicates the established LoQ for relative Rluc_Bb_ units. Black symbols represent extracts with quantifiable relative Rluc_Bb_ units and red symbols represent extracts with non-quantifiable Rluc_Bb_ units. A nymph extract with no detectable CFU (CFU = 0) is represented as the data point on the Y-axis. A significant positive correlation was detected between CFU and relative Rluc_Bb_ units (Pearson coefficient, *r* = 0.8022, *p* = 0.0017).

Based on our quantitation of the average number of spirochetes per tick (Figure 4), we estimated that pools of 24 fed larvae would equate to approximately 10^4^ spirochetes per sample, suggesting that the Rluc_Bb_ activity would be quantifiable by our assay (Figure 2B). Therefore, 2 weeks following the blood meal, 24 fed larvae per experimental group, in triplicate, were crushed in PBS and relative Fluc_Bb_ and Rluc_Bb_ units measured using the luciferin and coelenterazine substrates, respectively (Table 2). The remaining fed larvae were reserved and allowed to molt into nymphs. The unfed, infected nymphs were then fed to repletion on naïve mice. Approximately 2 weeks post feeding, groups of eight fed nymphs were crushed in PBS, in triplicate, and assessed for relative Fluc_Bb_ and Rluc_Bb_ units (Table 2). Under the assumption that the spirochete load per fed nymph is increased approximately 10-fold compared to that of fed larvae (Jewett et al., 2007, 2009), we estimated the average spirochete load per fed nymph to be approximately 10^4^. Therefore, a pool of eight fed nymphs was estimated to equate to approximately 8 × 10^4^ spirochetes, which is above both the LoD and LoQ of the *in vitro* assay (Figure 2B). The actual LoQ for the *in vivo* tick assay was established using the average Rluc_Bb_ units plus 10 standard deviations for tick extracts from fed ticks infected with *B. burgdorferi* lacking *rluc*_Bb_ but expressing *flaBp*-*fluc*_Bb_, (+pJSB175), rather than PBS alone. This is due to the observation that this tick extract negative control resulted in lower background relative Rluc_Bb_ units compared to PBS alone (Table 2). In contrast, there was no observed difference in the background relative Fluc_Bb_ units between PBS and the tick samples containing *B. burgdorferi* with a promoterless *fluc*_Bb_ and expressing *flaBp*-*rluc*_Bb_ (+pCFA701) in the luciferin assay. Therefore, the LoQ for Fluc_Bb_ in the *in vivo* tick assay was determined using the average relative Fluc_Bb_ units for PBS plus 10 standard deviations. Samples that fell below the LoQ threshold for either luciferase enzyme were considered no different than background (Table 2). As expected, we detected quantifiable relative Rluc_Bb_ units for all fed larvae samples, and all but two fed nymph samples (Table 2), indicating that sufficient spirochetes were present in the samples for the assay. In the pools of fed larvae only samples containing *B. burgdorferi* carrying *flaBp-fluc*_*Bb*_ (+pCFA801) demonstrated quantifiable relative Fluc_Bb_ units. The activities of *ospAp* and *ospCp* were below the LoQ for Fluc_Bb_ (Table 2). In contrast, all three promoters produced quantifiable relative Fluc_Bb_ units in the fed nymphs. Although one of the extracts from the fed nymphs infected with *B. burgdorferi* carrying *ospCp-fluc*_*Bb*_ (+pCFA803) did not result in quantifiable relative Fluc_Bb_ units, this sample also failed to achieve quantifiable relative Rluc_Bb_ units (Table 2), indicating that the number of spirochetes in the sample was insufficient for the assay. The promoter activities of the spirochetes in the fed nymph samples were analyzed by subtracting the average relative Fluc_Bb_ units of PBS from the relative Fluc_Bb_ units of each sample and the average relative Rluc_Bb_units of the infected tick extracts containing the negative control plasmid (+pJSB175) from the relative Rluc_Bb_ units of each sample. Background-subtracted Fluc_Bb_ units were then normalized to the respective background-subtracted relative Rluc_Bb_ units, for all quantifiable values. The Rluc_Bb_-normalized promoter activities reflected the expected corresponding *B. burgdorferi* transcript expression pattern during the fed nymph life stage (Figure 5A, Iyer et al., 2015).

**Table 2:** *In vivo* tick dual luciferase assay.

**Tick life stage**	**Plasmid**	**Luciferase cassette(s)**	**Relative Rluc**_**Bb**_ **units/biological replicate**^**a**^			**Relative Fluc**_**Bb**_ **units/biological replicate**^**b**^		
			**1**	**2**	**3**	**1**	**2**	**3**
Fed Larvae^e^	PBS	n/a^c^	85.2	89.6	92.8	20.0	24.8	24.4
	pJSB175	*flaBp-fluc_Bb_*	37.6	36.4	34.4	137.6	245.6	138
			LoQ^d^ = 52.3			LoQ = 49.7		
	pCFA701	*flaBp-rluc_Bb_*	185.6	108.8	208.0	22.4^*^	24.0^*^	24.0^*^
	pCFA801	*flaBp-rluc_Bb_; flaBp-fluc_Bb_*	603.6	1,502.4	1,187.6	56.0	136.0	100.8
	pCFA802	*flaBp-rluc_Bb_; ospAp-fluc_Bb_*	144.4	153.2	176.0	26.0^*^	25.2^*^	24.0^*^
	pCFA803	*flaBp-rluc_Bb_; ospCp-fluc_Bb_*	346.8	341.2	234.8	22.4^*^	23.6^*^	19.6^*^
Fed Nymph^f^	PBS	n/a	82.4	93.2	83.2	19.6	20.4	18.8
	pJSB175	*flaBp-fluc_Bb_*	31.6	31.6	30.0	504.8	1,700.8	881.6
			LoQ = 40.3			LoQ = 27.6		
	pCFA701	*flaBp-rluc_Bb_*	41.6	29.6^*^	296.0	18.0^*^	19.6^*^	18.0^*^
	pCFA801	*flaBp-rluc_Bb_; flaBp-fluc_Bb_*	2,416.8	2,560.0	780.4	956	552.8	388
	pCFA802	*flaBp-rluc_Bb_; ospAp-fluc_Bb_*	1,001.2	2,257.6	338.4	107.6	262	36
	pCFA803	*flaBp-rluc_Bb_; ospCp-fluc_Bb_*	1,500.8	1,239.6	34.8^*^	21.2^*^	32.8	16.4^*^

a *Relative Rluc_Bb_ units from three independent tick extracts incubated with 3.5 mM coelenterazine*.

b *Relative Fluc_Bb_ units from three independent tick extracts incubated with 700 μM luciferin*.

c *Not applicable*.

d *Limit of Quantification (LoQ) defined as the average background signal for each assay plus 10 standard deviations*.

e *Extract from groups of 24 fed larvae crushed in PBS*.

f *Extract from groups of 8 fed nymphs crushed in PBS*.

* *Samples that fell below their respective LoQ*.

As an additional means to validate the method as well as to demonstrate that relative Rluc_Bb_ units are directly reflective of spirochete numbers in the infected tick samples, a portion of each sample from the fed infected nymphs used for Rluc_Bb_ and Fluc_Bb_ quantitation (Table 2, Figure 5A), was plated in solid BSKII medium for determination of *B. burgdorferi* CFUs. The average CFUs per 100 μl of tick extract, the same volume used for the dual luciferase assay, across all clones, was found to be 3.72 × 10^5^ spirochetes. These data support our rationalization for the use of 8 fed nymphs in the assay. Raw relative Rluc_Bb_ units (Table 2) for these samples plotted against their corresponding CFU counts demonstrated a significant positive correlation (Figure 5B). Furthermore, this analysis indicated that 1.2 × 10^3^ spirochetes are sufficient to generate relative Rluc_Bb_ units above the LoQ for the *in vivo* tick assay, which is similar to the sensitivity we observed for the *in vitro* assay. Collectively, we have described a valuable new method to determine the activity of *B. burgdorferi* promoters of interest under *in vitro* growth conditions and in infected ticks. This is the first application of a dual reporter system for *B. burgdorferi* and, to the best of our knowledge, the first quantification of spirochete promoter activities in the tick vector.

## Discussion

Promoter fusion reporter systems are elegant, simple, and powerful tools to quantitate bacterial promoter activities in environments of interest. Herein we have established a new dual luciferase reporter method using the *Renilla* (sea pansy) and *Photinus* (firefly) luciferase enzymes for measurement of *B. burgdorferi* promoter activities *in vitro* and in the feeding tick during spirochete acquisition from an infected vertebrate host and transmission to a naïve vertebrate host. We demonstrate that constitutive expression of the *B. burgdorferi* codon-optimized *Renilla* luciferase gene (*rluc*_*Bb*_) is a specific and sensitive measurement of spirochete numbers for normalization of *Photinus* luciferase gene (*fluc*_*Bb*_) expression under the control of a promoter of interest.

Several reporter genes have been applied to *B. burgdorferi* including chloramphenicol acetyl transferase (*cat*) (Sohaskey et al., 1997), genes encoding a variety of fluorescent proteins (Eggers et al., 2002; Carroll et al., 2003; Schulze and Zuckert, 2006), the *Photinus pyralis* luciferase gene (*fluc*_*Bb*_) (Blevins et al., 2007), and *lacZ* encoding β-galactosidase (*lacZ*_*Bb*_) (Hayes et al., 2010). Here we describe the first use of a dual reporter system for *B. burgdorferi*. The combined application of the *Renilla* and *Photinus* luciferase genes has several advantages compared to other *B. burgdorferi* reporter systems as well as other methods of gene expression quantitation such as RT-qPCR. No sample extraction or purification is required to achieve detectable bioluminescence signals, allowing for rapid assay read out with little sample manipulation. Our data indicate that the *rluc*_*Bb*_ gene under the control of the strong, constitutive *flaB* promoter results in relative Rluc_Bb_ units reflective of the number of live spirochetes. This allows relative Rluc_Bb_ units to serve as the endogenous control against which the relative luciferase units of promoter fusions to *fluc*_*Bb*_ on the same plasmid, in the same sample, can be normalized. It is even possible to measure Fluc_Bb_ and Rluc_Bb_ signals back-to-back in the same assay well using firefly luciferase quenching reagents, such as Stop & Glo by Promega (McNabb et al., 2005) and therefore little sample material is required. Use of optical density at 600 nm (OD_600_) to quantitate sample turbidity as a measure of cell number does not distinguish between live and dead cells in the sample and therefore may not accurately reflect the number of live cells that contribute to the bioluminescence signal. Furthermore, OD_600_ cannot be used for complex biological samples such as extracts from fed ticks. We demonstrate a significant positive correlation between relative Rluc_Bb_ units and numbers of live spirochetes both *in vitro* and in ticks. The *B. burgdorferi* clone containing pCFA802 exhibited statistically different relative Rluc_Bb_ units *in vitro* when normalized to OD_600_ compared to the other clones. However, the relative Fluc_Bb_ units/10^8^ relative Rluc_Bb_ units for this clone followed the expected pattern of *ospA* expression *in vitro* and in nymphs. Furthermore, the relative Rluc_Bb_ units for spirochetes carrying pCFA802 correlated to the number of live spirochetes in fed nymph extracts from this clone, suggesting that the observed difference may not result in a biologically significant effect. Utilizing *flaBp-rluc*_Bb_ as an endogenous constitutive control provides new opportunities for the development of novel high-throughput genetic screening approaches. DNA libraries engineered to drive expression of *fluc*_Bb_ could be effectively screened for active promoters in various growth conditions of interest and relative Fluc_Bb_ units normalized to relative Rluc_Bb_ units. Further, the dual luciferase reporter plasmid can be manipulated to engineer Fluc_Bb_ translational fusions to quantitate protein production and stability in growth conditions of interest. An additional important benefit of the dual luciferase reporter assay is the ability to quantitate the promoter activity of a transcript in a strand-specific manner. We and others have recently reported recognition of novel RNA transcripts in the *B. burgdorferi* genome (Arnold et al., 2016; Adams et al., 2017; Popitsch et al., 2017). Through global 5′ end mapping of the *B. burgdorferi* transcriptome, we have predicted promoter sequences for previously unannotated RNAs, including antisense and intragenic transcripts, and validated their activities in a variety of environments (Adams et al., 2017). Application of the dual luciferase reporter system now provides a robust means for quantitative comparative analysis of strand-specific *B. burgdorferi* transcription in complex regions of the genome at the tick-pathogen interface.

For the correct interpretation of molecular techniques it is important to define the lowest level of a measurement, in this case relative luciferase units, which can be reliably analyzed. The limit of detection (LoD) is the lowest amount of measurable signal above background and the limit of quantification (LoQ) signifies the lowest interpretable signal above background. Effective use of LoD and LoQ are based off the standard deviation (SD) of background readings and assume at least 95% of analyzed values are true measurements in the biological assay (Armbruster and Pry, 2008). We have stringently defined LoD as the mean_background RLUs_+ 3SD and LoQ as the mean_background RLUs_ + 10SD. Thereby LoQ should be calculated for each luciferase substrate and each independent application of the *B. burgdorferi* dual luciferase assay to best distinguish low but quantifiable bioluminescence signals from background. It is also important to define the appropriate background controls in the context of the assay. Indeed, our studies have demonstrated that background relative Rluc_Bb_ units were ~60% decreased in fed tick extracts compared to PBS alone. Therefore, extracts from fed ticks infected with *B. burgdorferi* lacking *rluc*_Bb_ expression (+pJSB175) served as the background control to calculate the LoQ for Rluc_Bb_ in ticks. Conversely, this was not observed for the background relative Fluc_Bb_ units for fed tick extracts and PBS alone served as the negative control for these measurements. We hypothesize that the biological matrix of the fed tick extracts contributes, in part, to alteration of the Rluc_Bb_ signal by inhibiting non-specific activation of the coelenterazine substrate.

We found that not all samples with quantifiable relative Rluc_Bb_ units, also had quantifiable relative Fluc_Bb_ units. In some cases, the finding that a promoter fusion has non-quantifiable relative Fluc_Bb_ units may accurately reflect the weak to no biological activity of that promoter in a particular environment and/or non-quantifiable relative Fluc_Bb_ units may result from low numbers of spirochetes, albeit quantifiable relative Rluc_Bb_ units. These challenges may be overcome by increasing the number of spirochetes used in the assay. This is evident in the data we present for the *in vivo* tick assay, in which the fed larvae samples for all *B. burgdorferi* clones achieved quantifiable relative Rluc_Bb_ units; however, the clone containing *flaBp-fluc*_*Bb*_ (+pCFA801), but not the clones containing *ospAp-fluc*_*Bb*_ (+pCFA802) or the *ospCp-fluc*_*Bb*_ (+pCFA803), produced quantifiable relative Fluc_Bb_ units. This finding was not surprising for the *ospC* promoter, given that the *ospC* transcript is known to have weak to no activity in fed larvae following *B. burgdorferi* acquisition from infected mice. This finding was, however, unexpected for the *ospA* promoter, whose transcript is known to have strong activity in this environment (Caimano et al., 2015). Yet, the average number of spirochetes in the *ospAp-fluc*_*Bb*_ (+pCFA802) and *ospCp-fluc*_*Bb*_ (+pCFA803) containing clone extracts, as reflected by the average relative Rluc_Bb_ units (1.6 × 10^2^ and 3.1 × 10^2^, respectively), were approximately 10-fold and 4-fold less than that of the *flaBp-fluc*_*Bb*_ (+pCFA801) containing clone (1.1 × 10^3^), suggesting that spirochete number may contribute, in part, to the non-quantifiable relative Fluc_Bb_ units for these spirochetes. In contrast, the fed nymph extracts contained comparable average numbers of spirochetes regardless of the clone, as reflected by both the average relative Rluc_Bb_ units (1.5 × 10^3^ ± 380) and CFU counts (4.4 × 10^5^ ± 2.2 × 10^5^) and all *fluc*_*Bb*_ promoter fusions achieved quantifiable relative Fluc_Bb_ units. Furthermore, while it was one of our goals to measure promoter activities for spirochetes in unfed-flat nymphs post-molt, we found the luciferase signals for these samples to be below the limit of quantification of our assay. We again hypothesize that the spirochete loads in the ticks at this point in the infectious cycle may be below the number of spirochetes necessary for the assay. To examine this possibility we crushed and plated for CFU a subset of individual unfed nymphs infected with spirochetes carrying both *flaBp-rluc*_*Bb*_ and *flaBp-fluc*_*Bb*_ (pCFA801). The average spirochete load was determined to be ~27 spirochetes/unfed nymph. This was approximately 10-fold lower than the average spirochete load in the fed larval ticks for the same clone (~4.4 × 10^2^ spirochetes/fed larvae) and approximately 10,000-fold lower than that of fed nymphs (~1.6 × 10^5^ spirochetes/fed nymph). Considering that pools of 24 fed larvae and 8 fed nymphs, and therefore ~10^4^ and ~10^6^ spirochetes carrying pCFA801, respectively, were used for the luciferase assays, nearly 400 up to 40,000 unfed nymphs would be required to achieve equivalent relative luciferase units. The difficulties of studying *B. burgdorferi* transcription in unfed nymphs was also shown by a recent microarray study, where even with an amplification step, transcript analysis in this tick life-stage was precluded (Iyer et al., 2015). RT-qPCR does remain an alternative approach for gene expression analysis in unfed nymphs, having several documented successes in determining *B. burgdorferi* transcript levels (Wang et al., 2002; Bykowski et al., 2007; Showman et al., 2016), albeit lacking strand specificity. It should be noted that the endogenous copies of the *flaB, ospA*, and *ospC* genes and their promoters are present in the genetic background of all of the *B. burgdorferi* clones that were analyzed. This raises the possibility that a reduction in Fluc_Bb_ or Rluc_Bb_ signals could have occurred due to titration of transcription factors away from the promoter fusions by the endogenous promoters. However, expression of *flaB, ospA*, and *ospC* are essential for survival of *B. burgdorferi* throughout its infectious cycle (Samuels, 2011; Sultan et al., 2013) and thus these experiments could not be conducted in the absence of these genes.

While dual *fluc* and *rluc* reporter systems have been used successfully for live imaging and quantitation of eukaryotic tumor cells in mice (Bhaumik and Gambhir, 2002), the use of *Renilla* luciferase and the coelenterazine substrate for live imaging of microbial infections in mice has proven challenging (Andreu et al., 2011) and few publications report exploration of the use of dual *Renilla* and *Photinus* luciferase reporters in the context of infectious disease applications. There is great interest in applying a luciferase dual reporter system to quantification of *B. burgdorferi* promoter activities during an active mammalian infection. We and others have demonstrated the power of the *fluc*_*Bb*_ reporter for tracking *B. burgdorferi* dissemination and qualitative detection of promoter activities over time in live mice (Hyde et al., 2011; Chan et al., 2015; Adams et al., 2017). By extension we investigated the efficacy of the dual luciferase reporter system for live imaging applications with *B. burgdorferi* in infected mice. Exhaustive examination of available coelenterazine substrates including: h-Coelenterazine-SOL *in vivo* (NanoLight), Inject-A-Lume h-Coelenterazine (NanoLight), ViviRen™ *in vivo* Renilla Luciferase Substrate (Promega), and XenoLight RediJect Coelenterazine h (PerkinElmer) as well as various substrate concentrations, substrate injection methods and imaging times, resulted in no significant Rluc_Bb_ signals above background (data not shown). Unlike for applications for solid cancers, use of luciferase substrates for *in vivo* detection of microbial pathogens relies on the substrates to be available in excess, systemically throughout the animal. Luciferin has been documented to rapidly distribute throughout the mouse (Contag et al., 1997), but the bioavailability of coelenterazine may be more limited (Luker et al., 2002). In addition, we found coelenterazine to have an extraordinary high background signal. Indeed, Rluc_Bb_ signals following coelenterazine delivery were observed for mice infected with spirochetes lacking *rluc*_*Bb*_ entirely, which were not able to be overcome in mice infected with spirochetes expressing *flaBp-rluc*_*Bb*_ (data not shown). These findings are consistent with what has been reported for attempted *in vivo* imaging applications using coelenterazine and *Mycobacterium smegmatis* expressing *Gaussia* luciferase (Andreu et al., 2010). Rather, alternative methods of normalization may be used, such as determining spirochete loads of infected tissues immediately following Fluc_Bb_ imaging (Skare et al., 2016), in instances where quantification of promoter activity during murine infection is warranted.

*B. burgdorferi* has been shown to colonize *Ixodes scapularis* via a biphasic mode of dissemination which is believed to involve complex interactions between the pathogen and the arthropod vector (Dunham-Ems et al., 2009). We are still discovering many of the mechanisms *B. burgdorferi* employs to survive throughout its enzootic cycle. Additionally, the recently sequenced *Ixodes scapularis* genome opens new areas of study for host-pathogen interactions (Gulia-Nuss et al., 2016). Successful and reliable techniques for analysis of spirochete biology in the tick are critical to drive understanding of these interactions. The dual luciferase system presented here is a simple and powerful approach for measuring transcript expression, which can be easily modified to meet the needs of the researcher and adds to the ever growing molecular genetic toolbox for investigation of *B. burgdorferi* transcription and gene regulation.

## Author contributions

PA and MJ conceived the study and designed experiments; PA and CF performed experiments; PA, CF, and MJ interpreted results; PA and MJ wrote the manuscript; all authors critiqued and edited the final manuscript.

### Conflict of interest statement

The authors declare that the research was conducted in the absence of any commercial or financial relationships that could be construed as a potential conflict of interest.
